# Synthesis of functional polyacrylamide (co)polymers by organocatalyzed post-polymerization modification of non-activated esters

**DOI:** 10.1039/d3ra04667b

**Published:** 2023-10-03

**Authors:** Guoqing Li, Zhiyi Zhang, Wenhao Xiao, Tongtong Wu, Jinbao Xu

**Affiliations:** a Jiaxing Key Laboratory of Preparation and Application of Advanced Materials for Energy Conservation and Emission Reduction, College of Advanced Materials Engineering, Jiaxing Nanhu University Jiaxing 314001 P. R. China ligq@jxnhu.edu.cn; b Jiangxi General Institute of Testing and Certification Nanchang 330052 P. R. China; c Guangdong Provincial Key Laboratory of Functional Soft Condensed Matter, School of Materials and Energy, Guangdong University of Technology Guangzhou 510006 P. R. China xujinbao@gdut.edu.cn

## Abstract

The broad application of polyacrylamides (PAMs) has greatly promoted the development of new synthetic methods to prepare PAM-based functional (co)polymers regarding their traditional preparation *via* the direct polymerization of various acrylamide monomers. Herein, we have explored the post-polymerization modification of the poly(2,2,2-trifluoroethyl acrylate) (PTFEA) homopolymer, a typical non-activated ester, and various amines using the organo-catalytic system involving 1,8-diazabicyclo[5.4.0]undec-7-ene (DBU) and 1,2,4-triazole (TA). The reaction kinetics (*e.g.*, the optimized reaction solvent, temperature, time, initial molar ratio of amines to esters and the molar ratio of DBU to TA) were carefully studied with the modulus substrate of iso-propylamine as the formed poly(iso-propyl acrylamide) (PNIPAM) representing the most investigated PAM. The full and partial amidation of the esters in PTFEA could be precisely regulated just by controlling the kinetic conditions to give (co)polymers with designable compositions and structures. We have demonstrated that the poly(*N*-acryloyl pyrrolidine) obtained by the post-polymerization modification of non-activated ester and pyrrolidine exhibited a noticeable phase transition, which confirmed the robustness and versatility of the post-polymerization modification. The described method paves the way for the synthesis of various (co)polymers with amide side chains from readily available polymer precursors.

## Abstract 2

Synthesis of functional polyacrylamide (co)polymers by organocatlyted post-polymerization modification of poly(2,2,2-trifluoroethyl acrylate).

## Graphical abstract

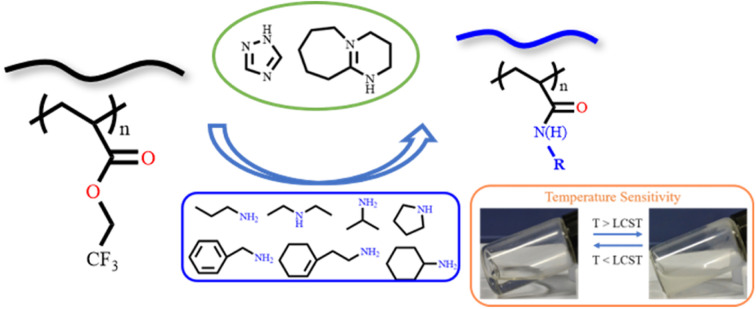

## 1. Introduction

The constant enrichment of the design and fabrication of functional (co)polymers has benefited from the development of novel polymerization mechanisms, and protocols have flourished with their utilization, ranging from common household items (*e.g.*, kitchen appliances, toiletries and cosmetics) to advanced applications, such as biomedical materials, energy storage devices, aerospace materials, *etc.* Post-polymerization modification reactions (generally the reaction between functional small molecules and macromolecular precursors) have recently been extensively studied and represent the most attractive protocol for preparing novel functional macromolecules, considering the straightforward and versatile regulation of polymer compositions, architectures and functionalities,^1–4^ while partly overcoming the disadvantages in traditional polymerizations, *i.e.*, the incompatibility of functional monomers and polymerization protocols, such as the reservation of pendant unsaturated bonds in free-radical polymerization (FRP) and the direct preparation of hetero-functional extremities in ring-opening polymerization (ROP) (*e.g.*, the coexistence of hydroxyl and amine groups).^5–8^ Particularly, the rapid emergence of high-efficiency protocols based on click chemistry (*e.g.*, the cycloaddition of azide–alkyne and Diels–Alder reactions, thiol–ene/–yne additions, halogens and carboxylic acids, transformation reactions of esters and amines/alcohols) has dramatically accelerated the development of post-polymerization modification reactions.^9–14^ Among the protocols mentioned above, transformation reactions of esters and amines/alcohols have received continuous attention regarding the versatility and robustness in synthesizing polyacrylamide (PAM) (co)polymers, which are among the most utilized macromolecules; for example, in the field of wastewater treatment, soil decontamination and biomedicine, *etc.*^15–17^

Several pre-designed macromolecular precursors embodying activated esters or amides, for example, poly(*N*-hydroxysuccinimide (meth)acrylates),^18–20^ poly(pentafluoro phenyl (meth)acrylates),^21–23^ poly(di-Boc acrylamides),^24–26^ and poly(alkyl salicylate acrylates),^27–29^ have been utilized as plausible substrates for transformation reactions between ester and amines/alcohols for the preparation of functional materials with expected compositions and architectures. Compared with the pronounced studies focused on the post-polymerization modification reactions of activated esters, non-activated esters, commonly synthesized through the direct (co)polymerization of commercially available (co)monomers (avoiding the essential modification process of monomers before polymerization), are still less explored in terms of the poor reaction kinetics of functional amines or alcohols. Although some research groups have demonstrated the successful transformation of non-activated esters and amines/alcohols, *e.g.*, poly(meth)acrylates,^30–37^ poly(2-oxazoline)s,^38–43^ understanding the reaction kinetics and the relationship between the polymer structure and properties of non-activated esters is still of great interest.

**Scheme 1:**
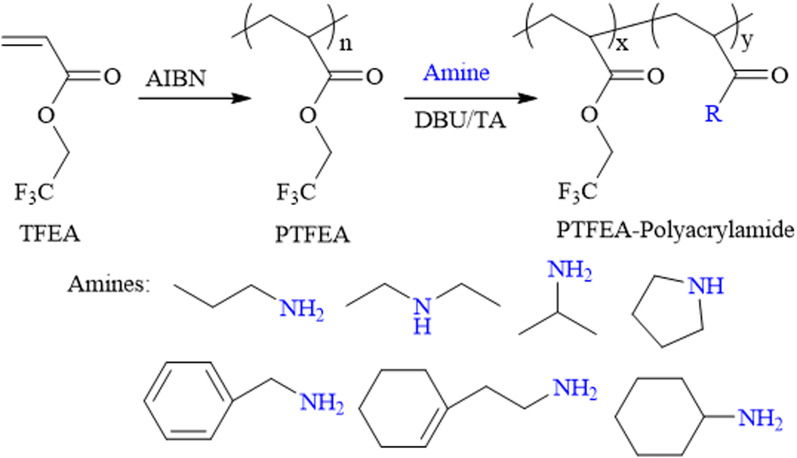
Schematic illustration of the transformation reaction of esters and amines.

Theato *et al.* reported the first example of the activation of non-activated esters including poly(2,2,2-trifluoroethyl methacrylate) (PTFEMA) and poly(methyl methacrylate) (PMMA) in the presence of the 1,8-diazabicyclo[5.4.0]undec-7-ene (DBU) and 1,2,4-triazole (TA) co-catalytic system.^33^ Herein, the non-activated ester poly(2,2,2-trifluoroethyl acrylate) (PTFEA) was chosen as the model ester with DBU and TA being the promoters to systematically explore the details (*e.g.*, the reaction kinetics, the influence of solvent, and reaction time, the initial molar ratio of amines to esters, the scope of amine) in the transformation of stable ester with a variety of functional primary or secondary amines (Scheme 1). Based on the exploration, the (co)polymers of PAMs were prepared and one thermosensitive homopolymer of poly(*N*-acryloyl pyrrolidine) was also prepared. We envision that this research could facilitate the preparation of PAM-based materials and advance the understanding of the transformation reaction between non-activated esters and amines.

## 2. Results and discussion

### 2.1. Studying the reaction kinetics of the post-polymerization between PTFEA and amines

**Fig. 1:**
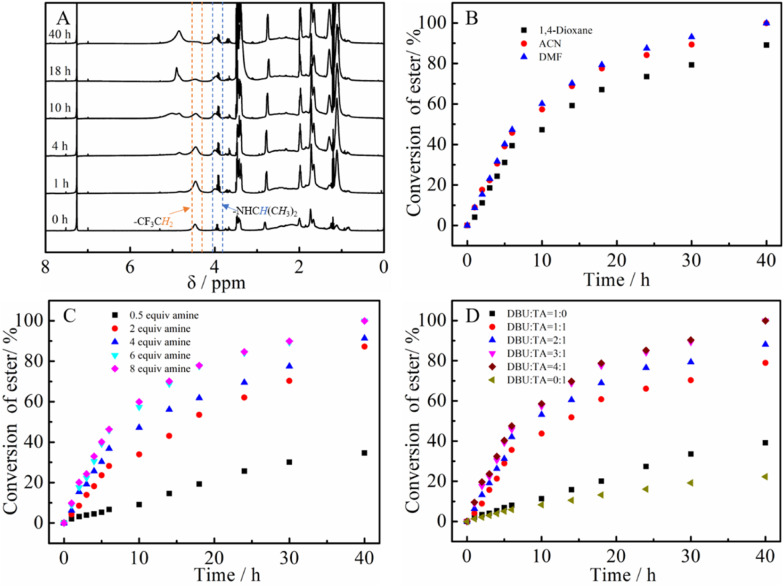
Kinetics studies of the post-polymerization modification of PTFAE and iso-propylamine: (A) the evolution of the ^1^H NMR spectra with time, (B) screening of solvents, the influence of the initial molar ratio of (C) amine to esters and (D) DBU to TA on the reaction.

The PTFEA macromolecular precursor was firstly synthesized by traditional FRP, given its non-activated ester features (the p*K*_a_ value is ∼23.5 in DMSO for CF_3_CH_2_OH^44^) as well as structure similarity after post-polymerization reactions with amines to PAMs. Although the uncontrollable polymerization process for traditional FRP generally suffers from high values of *Đ* in comparison with controllable FRP (*e.g.*, ATRP and RAFT), the disadvantage was preferably utilized herein to further demonstrate the robustness and versatility of the protocol. Iso-propylamine was selected as the substrate to evaluate the reaction kinetics of the post-polymerization reactions in the presence of the TA and DBU co-catalytic system due to the feasible detection of isopropyl groups in ^1^H NMR spectra as well as the distinguishable signal position from that of the precursor. Samples were taken at regular time intervals during the kinetic experiments and analyzed by ^1^H NMR to calculate the conversion of the esters (Fig. 1A). Remarkably, the peak intensity attributed to the methylene protons of –CF_3_CH_2_ showed a gradual decrease alongside the reaction times (almost negligible after 40 h) while the intensity of newly formed signals resulting from the methine and methyl protons of –NHCH(CH_3_)_2_ increased with the increasing reaction times. Additionally, the appearance of a signal assigned to the proton from –NHCH(CH_3_)_2_ indicated the formation of amide bonds in the post-polymerization reaction.

We then focused on the screening of the reaction conditions, such as solvent, temperature, time, catalyst loading, *etc.*, to achieve high efficiency of ester conversion. Fig. 1B shows the influence of solvent on the conversion of esters, and three kinds of solvents (1,4-dioxane, ACN and DMF) with different polarities were explored. The conversion of esters using DMF as the solvent was the highest, while that using 1,4-dioxane was the lowest for the same times, which is due to the better solubility of the formed PNIPAM in highly polar solvents. However, considering the higher boiling point of DMF that makes the removal process difficult, ACN was used as the solvent (the conversion of ester is a little lower than that in DMF). From Fig. 1B we also could conclude that the esters were almost transformed into amides within 40 h with iso-propylamine.

According to Theato's publication,^33^ the initial molar ratio of DBU to TA plays a crucial role in determining the final conversion of esters to amides. Fig. 1C displays the influence of the initial molar ratio of amine to esters on the conversion of esters. It is noticeable that the conversion of esters in the presence of only DBU or TA seems particularly low (∼36% for DBU and ∼20% for TA). When DBU and TA were both added, the conversion increased sharply even with a ratio of DBU/TA = 1/1. Attractively, the conversions of esters showed an increment with increasing the ratio of DBU to TA, whereas the conversion of esters for 4 : 1 and 3 : 1 seemed the same, indicating the optimal ratio of 3 : 1 for DBU/TA. After that, the influence of the initial ratio of DBU to TA was also estimated (Fig. 1D). Similar to the phenomenon in the screening of the DBU to TA ratio, the conversion of esters showed an increment with increasing the amine until a 6 : 1 ratio of amine to esters was reached. Although the conversion of esters increased with increasing the temperature (the data is not shown), room temperature was chosen as the reaction temperature due to the lower boiling point and instability of some amines at elevated temperatures. Based on the above results, the optimized reaction conditions for the post-polymerization of PTFEA with iso-propylamine are as follows: the initial ratio of esters was DBU : TA : amine = 1 : 3 : 1 : 6 with ACN solvent and the initial concentration of ester was 1.0 mol mL^−1^, which are selected reaction conditions for subsequent experiments.

### 2.2. Evaluating the scope of amines and preparing the (co)polymers of PAMs

Based on the reaction conditions established above, we then continued to explore the diversity of the DBU/TA catalytic post-polymerization modification of non-activated esters, aiming to prepare various PAAs. Table 1 summarizes the results of the obtained samples after the transformation reaction of amines and PTFEA. Besides iso-propylamine, six commercially available amines were also tested, including primary (propylamine, cyclohexylamine, benzylamine and 1-cyclohexen-1-ylethylamine), secondary (diethyl amine and pyrrolidine), aromatic (benzylamine) and cyclic amines (pyrrolidine), where the difference exists in the basicity, nucleophilicity and steric hindrance. Remarkably, the conversion of esters for the amines was higher than 97%, except for the benzylamine (∼89%) under our optimized conditions, suggesting the substrate diversity of the DBU/TA co-catalytic system for non-activated esters with quantified transformation. Moreover, there seemed to be no other side reactions, such as cross-linking, as the values of dispersity (*Đ*) of the isolated polymers showed little changes.

**Table 1:** Results of the post-polymerization modification with various amines

Runs^a^	Amines	*M*_n_,_SEC_ × 10^−4^^b^ (g mol^−1^)	*Đ* b	Conversion of ester^c^ (%)
1	Iso-propylamine	0.94	1.45	99
2	Propylamine	1.00	1.41	99
3	*N*,*N*-Diethylamine	1.20	1.38	99
4	Pyrrolidine	1.15	1.48	99
5	Cyclohexylamine	1.40	1.41	97
6	Benzylamine	1.50	1.49	89
7	1-Cyclohexen-1-ylethylamine	1.95	1.36	98

a Reaction conditions: 6 equiv. of amine to esters, DBU : TA = 3 : 1 (molar ratio) in ACN at room temperature for 48 h.

b Determined by SEC.

c Determined by ^1^H NMR.

**Fig. 2:**
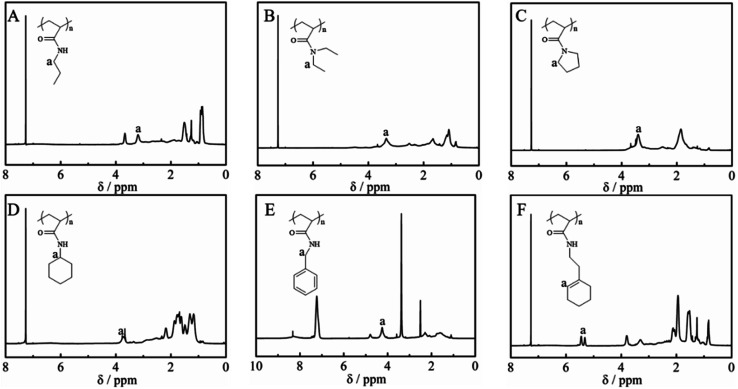
^1^H NMR spectra of (A) poly(*N*-propyl acrylamide), (B) poly(*N*,*N*-diethyl acrylamide), (C) poly(acryloyl pyrrolidine), (D) poly(*N*-cyclohexyl acrylamide), (E) poly(*N*-benzyl acrylamide) and (F) poly(*N*-[(1-cyclohexen-1-yl)ethyl]-2-acrylamide).

Fig. 2 shows the ^1^H NMR spectra of the purified polymers after post-polymerization with various amines. The proton signals located at 3.20, 3.35, 3.40, 3.72, 4.24 and 5.31 ppm were assigned to the typical peaks of the methylene protons adjacent to the amide (–NHC*H*_2_CH_2_CH_3_) from poly(*N*-propyl acrylamide) (Fig. 2A), the methylene protons of the ethyl group (–N(C*H*_2_CH_3_)_2_) from poly(*N*,*N*-diethyl acrylamide) (Fig. 2B), the methylene protons adjacent to the nitrogen atom from poly(*N*-acryloyl pyrrolidine) (Fig. 2C), the methine proton of the cyclohexyl group from poly(*N*-cyclohexyl acrylamide) (Fig. 2D), the methylene protons of the benzyl group (–NHC*H*_2_Ph) from poly(*N*-benzyl acrylamide) (Fig. 2E) and the double bond proton of cyclohexene from poly(*N*-[(1-cyclohexen-1-yl)ethyl]-2-acrylamide) (Fig. 2F). Although it is generally considered that the amidation between non-activated esters and amines by post-polymerization modification may be incomplete due to the macromolecular structure, the selected amines herein all performed with high efficiency to transform the esters in PTFEA, where the formed trifluoroethanol and the DBU/TA co-catalysts promoted the transformation.

**Fig. 3:**
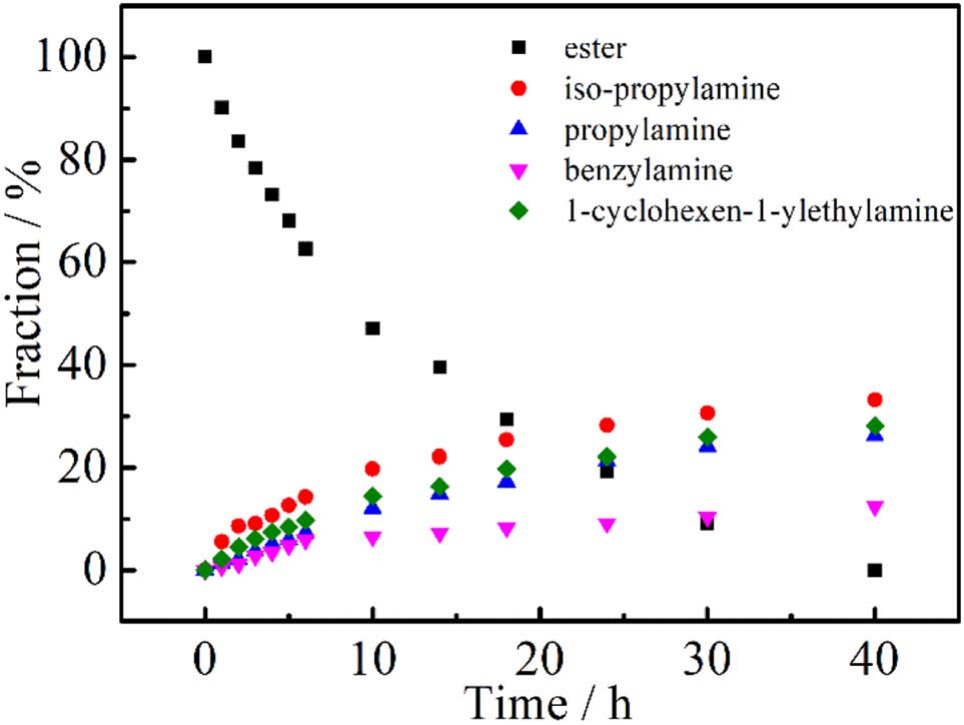
Evaluation of the esters and amide fractions with time for the post-polymerization of PTFEA with an equimolar mixture of iso-propylamine, propylamine, benzylamine and 1-cyclohexen-1-ylethylamine, performed with 3 equiv. DBU, 1 equiv. TA and a total of 6 equiv. amines.

It is well known that the physical and chemical properties of PAMs are heavily dependent on the composition and structure of the macromolecules. Particularly, the lower critical solution temperature (LCST) or upper critical solution temperature (UCST) could be finely mediated by introducing hydrophilic or hydrophobic segments to PAMs, for example, the most studied one, PNIPAM.^45^ We then tried to prepare (co)polymers based on PNIPAM to further demonstrate the robustness of the DBU/TA co-catalytic post-polymerization modification of the non-activated ester. Firstly, equimolar amounts of iso-propylamine, propylamine, benzylamine and 1-cyclohexen-1-ylethylamine were added (the total amount of amines to ester was also 6 : 1) to conduct the post-polymerization reaction, considering the different nucleophilicities of the selected amines. As shown in Fig. 3, the fraction of ester groups decreased with the reaction time, while iso-propylamine showed a faster rate of incorporation than the less nucleophilic propylamine, benzylamine and 1-cyclohexen-1-ylethylamine,^34,46^ indicating the plausible preparation of PNIPAM-based (co)polymers using DBU/TA catalytic non-activated transformation of esters to acrylamides.

**Scheme 2:**
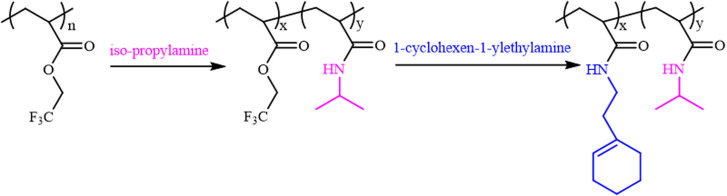
The preparation of PNIPAM-based PAMs.

Regarding the high-efficiency transformation of esters to amides with iso-propylamine, it is possible to precisely regulate the composition of PNIPAM-based PAMs through post-polymerization modification. As shown in Scheme 2, the amidation was firstly carried out between PTFEA and iso-propylamine and then 1-cyclohexen-1-ylethylamine was used to transform the residual esters, where the double bonds could be further utilized. Based on the amidation kinetics of iso-propylamine to esters, the transformation of esters to iso-propyl acrylamides could be finely designed, depending on the reaction time.

**Fig. 4:**
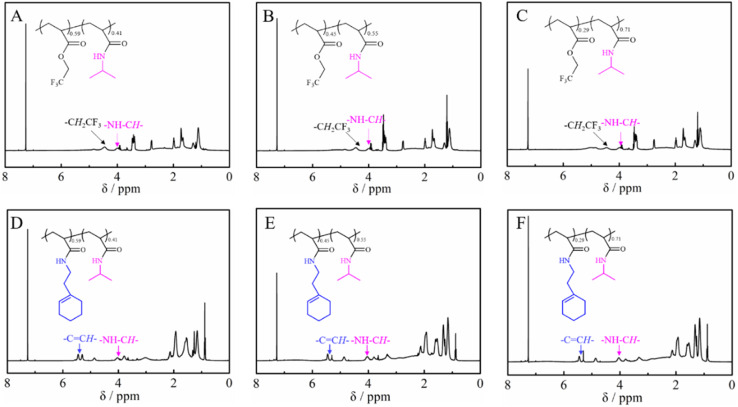
^1^H NMR of (A) PTFAE_0.59_-*co*-PNIPAM_0.41_, (B) PTFAE_0.45_-*co*-PNIPAM_0.55_, (C) PTFAE_0.29_-*co*-PNIPAM_0.71_, (D) poly(*N*-[(1-cyclohexen-1-yl)ethyl]-2-acrylamide)_0.59_-*co*-PNIPAM_0.41_, (E) poly(*N*-[(1-cyclohexen-1-yl)ethyl]-2-acrylamide)_0.45_-*co*-PNIPAM_0.55_ and (E) poly(*N*-[(1-cyclohexen-1-yl)ethyl]-2-acrylamide)_0.29_-*co*-PNIPAM_0.71_.

Tables 2 and 3 summarize the results of the obtained PTFEA-*co*-PNIPAM and poly(*N*-[(1-cyclohexen-1-yl)ethyl]-2-acrylamide)-*co*-PNIPAM (co)polymers, respectively. As shown in Fig. 4A–C, the conversion of esters can reach ∼41%, 55% and 71% with reaction times of 4 h, 6 h and 14 h, respectively. However, the *Đ* values of the PAMs increased from 1.21 for PTFEA to around 1.40 for PTFEA-*co*-PNIPAM, which is due to the interaction between the column and PNIPAM. After the introduction of 1-cyclohexen-1-ylethylamine, the signal due to the methylene protons of the trifluoro ethyl groups around 4.47 ppm completely disappeared according to Fig. 4D–F, while the typical signal of double bonds from poly(*N*-[(1-cyclohexen-1-yl)ethyl]-2-acrylamide) appeared at 5.31 ppm. Thus, we could prepare PNIPAM-based PAMs from the non-activated esters by partial or full amidation and the composition and structure could be finely regulated. In addition, considering the wide scope of the utilized amines with DBU/TA co-catalysts, we envision that a variety of functional PAMs could be facilely synthesized.

**Table 2:** Preparation of PTFAE-*co*-PNIPAM

Sample^a^	Time (h)	*M*_n_,_SEC_ × 10^−4^^b^ (g mol^−1^)	*Đ* b	Conversion of esters^c^ (%)
PTFAE_0.59_-*co*-PNIPAM_0.41_	4	1.40	1.45	41
PTFAE_0.45_-*co*-PNIPAM_0.55_	6	1.29	1.40	55
PTFAE_0.29_-*co*-PNIPAM_0.71_	14	1.16	1.43	71

a Reaction conditions: 6 equiv. of amine to esters, DBU : TA = 3 : 1 (molar ratio) in ACN at room temperature for 48 h.

b Determined by SEC.

c Determined by^1^H NMR.

**Table 3:** Preparation of poly(*N*-[(1-cyclohexen-1-yl)ethyl]-2-acrylamide)-*co*-PNIPAM

Sample^a^	*M*_n_,_SEC_ × 10^−4^^b^ (g mol^−1^)	*Đ* b	Conversion of ester^c^ (%)	
			*z* (%)	*y* (%)
Poly(*N*-[(1-cyclohexen-1-yl)ethyl]-2-acrylamide)_0.59_-*co*-PNIPAM_0.41_	1.76	1.42	59	41
Poly(*N*-[(1-cyclohexen-1-yl)ethyl]-2-acrylamide)_0.45_-*co*-PNIPAM_0.55_	1.52	1.42	45	55
Poly(*N*-[(1-cyclohexen-1-yl)ethyl]-2-acrylamide)_0.29_-*co*-PNIPAM_0.71_	1.24	1.41	29	71

a Reaction conditions: 6 equiv. of amine to esters, DBU : TA = 3 : 1 (molar ratio) in ACN at room temperature for 48 h.

b Determined by SEC.

c Determined by ^1^H NMR.

### 2.3. Evaluating the scope of amines and preparing the (co)polymers of PAMs

**Fig. 5:**
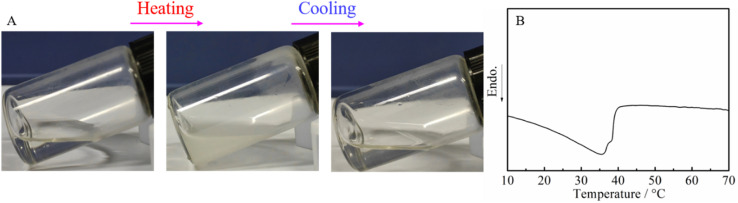
(A) Illustration of the phase transition upon heating and cooling the aqueous solutions of poly(*N*-acryloyl pyrrolidine) obtained from the post-polymerization reaction (25 to 40 °C), and (B) the DSC thermogram.

We finally focused on the application of the post-polymerization modification of the DBU/TA-catalyzed amidation of non-activated esters and amines. Poly(*N*-acryloyl pyrrolidine), a temperature-sensitive PAM with the LCST of around 50 °C,^47^ prepared through the FRP of *N*-acryloyl pyrrolidine that is generally synthesized with pyrrolidine and acryloyl chloride (highly corrosive chemical),^48^ was therefore prepared by the amidation of pyrrolidine and PTFEA herein to overcome the disadvantages of the traditional synthesis method. It is well known that phase separation is derived from the disparity between the dissolved polymers and solvents or the hydrophobic interaction.^49^ As shown in Fig. 5A, the aqueous solution of poly(*N*-acryloyl pyrrolidine) was transparent at room temperature and became an opaque solution by gradually elevating the solution temperature, which confirmed the occurrence of phase separation for the tested polymer. Attractively, the solution turned transparent again by gradually decreasing the temperature, indicating the reversible thermos-responsive phase transition. The phase transition due to the loss of the specific hydrogen bonds is an endothermic process, where the transition can be measured by calorimetric methods, *e.g.*, DSC.^50–52^ An endothermic peak was observed on the DSC curve (Fig. 5B) of the polymer obtained from the post-polymerization reaction, which indicated that the phase separation process undoubtedly occurs at this stage.

**Fig. 6:**
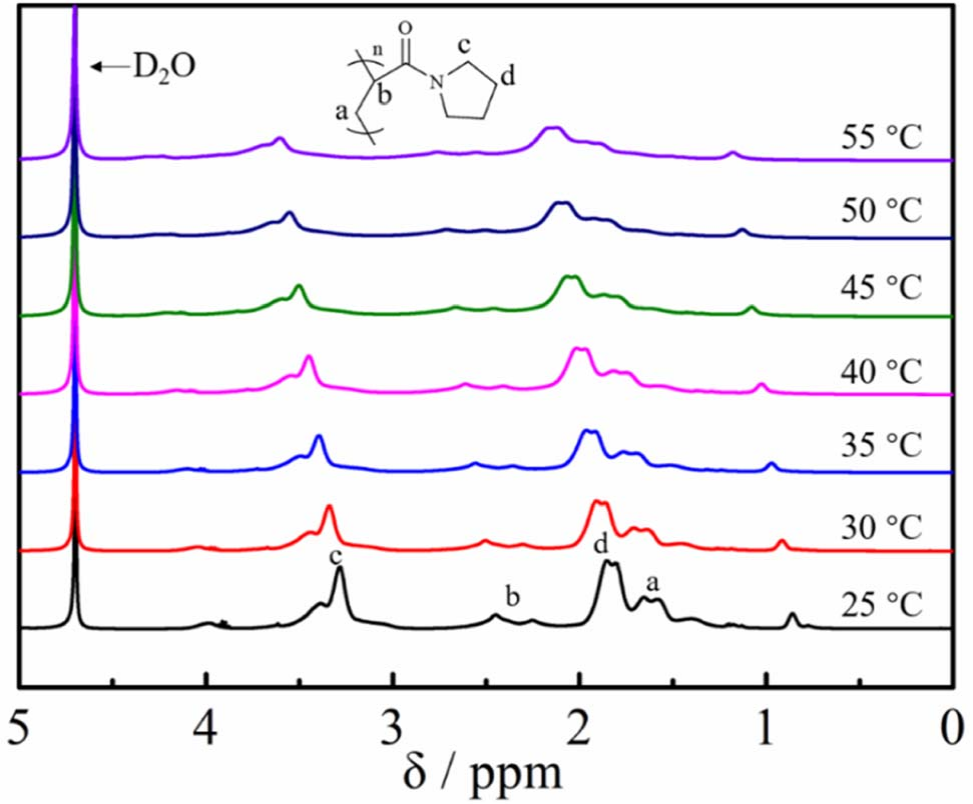
Temperature-variation ^1^H NMR spectral studies (in D_2_O) of poly(*N*-acryloyl pyrrolidine) obtained by post-polymerization modification.

To further investigate the temperature sensitivity behavior of the obtained poly(*N*-acryloyl pyrrolidine), a temperature variation ^1^H-NMR study was carried out. The proton signals of a molecularly dissolved polymer in the solvent can be detected and show a noticeable attenuation upon desolvation and can even collapse (after phase transition) when the solution temperature is increased.^53^ As shown in Fig. 6, the solvation/desolvation processes were well monitored by the temperature-variation ^1^H NMR spectra in deuterated water (D_2_O). Poly(*N*-acryloyl pyrrolidine) isolated by the post-polymerization reaction was initially dissolved molecularly in D_2_O at a low temperature, *e.g.*, 25 °C and the ^1^H NMR spectrum displays the typical proton resonance signals. As expected, upon elevating the solution temperature, the signals of the polymer showed a discernible down-field shift and the attenuation phenomenon could also be observed, undoubtedly testifying to the desolvation process (the phase transition) of the polymer by elevating the temperature. In addition, the ^1^H NMR signal intensity gradually increased with the slow cooling of the same solution (data not shown). The results from DSC and temperature-variation ^1^H NMR investigations indisputably corroborate the versatile and robust preparation of PAMs by the DBU/TA co-catalytic amidation of non-activated esters. Further studies on the detailed investigation of the phase transition behaviors of various functional PAMs and their applications are in progress in our lab.

## 3. Conclusion

In summary, we have surveyed the reaction kinetics for the DBU/TA co-catalytic post-polymerization modification of the non-activated ester of PTFEA and iso-propylamine and demonstrated that the esters could be fully or partially transformed into iso-propyl acrylamide in a controllable way based on the reaction kinetics. PNIPAM-based functional PAMs with pendant double bonds were also successfully synthesized by the straightforward post-polymerization modification reaction. The poly(*N*-acryloyl pyrrolidine) prepared by the amidation of PTFEA and pyrrolidine showed distinguishable phase transition behavior according to the DSC and temperature-variation ^1^H NMR characterizations. Therefore, the DBU/TA co-catalyzed transformation of non-activated esters to acrylamide seems to be a plausible protocol to directly prepare diverse PAMs (co)polymers.

## 4. Experimental section

### 4.1. Materials

2,2,2-Trifluoroethyl acrylate (TFEA) from Aldrich was passed through a column filled with basic aluminum oxide before use. Azodiisobutyronitrile (AIBN) from Aladdin was recrystallized with ethanol three times and stored at −10 °C. Tetrahydrofuran (THF) (Tianjin Chemical Reagent Co., Ltd China) was freshly distilled from sodium/benzophenone mixture under a protective nitrogen atmosphere. Other reagents from Aladdin or TCI were used as received.

### 4.2. Characterizations

A Bruker AV400 NMR spectrometer was used to analyze the proton nuclear magnetic resonance (^1^H NMR) of the samples while deuterated chloroform (CDCl_3_) or dimethyl sulfoxide (DMSO-*d*_6_) was used as the solvent with tetramethylsilane (TMS) as the internal standard. The apparent number-average molecular weight (*M*_n,SEC_) and *Đ* of the purified sample precursors were recorded on a size exclusion chromatograph (SEC, Waters) equipped with a 1515 pump and a 2414 differential refractive index detector with THF or dimethylformamide (DMF) (Aladdin, HPLC) eluent at a flow rate of 1.0 mL min^−1^ (35 °C). All the samples were dissolved in the eluent at a concentration of 5.0 mg mL^−1^ and then a 0.40 μm hydrophobic membrane was used to filter the solution. The experimental molecular weights were calculated according to a standard curve using a series of monodisperse polystyrenes as the standard samples. Fourier-transform infrared (FT-IR) spectra were recorded on an FT-IR-Digilab FTS3100 spectrometer with potassium bromide (KBr) pellets. Differential scanning calorimetry (DSC) was conducted on a Mettler Toledo DCS_3_ machine under a nitrogen flow of 50 mL min^−1^. The experimental temperature was lowered to 5 °C and maintained for another 5 min. Subsequently, it was reheated to 80 °C at a rate of 2 °C min^−1^ and the phase change temperature was defined as the transition center of the DSC curve.

### 4.3. The preparation of polymers

#### 4.3.1. Polymerization of PTFEA

The typical procedure for the traditional free-radical polymerization of TFEA using AIBN as the initiator is as follows. AIBN (53.3 mg, 0.325 mmol, 1 equiv.) and TFEA (5.0 g, 32.5 mmol, 100 equiv.) were added to a Schlenk reactor filled with 10 mL of purified THF. The solution was then degassed in three freeze–pump–thaw cycles before being placed into an oil bath at 70 °C. After polymerization for 10 h, the mixture was added to *n*-hexane dropwise and then the filtrate was collected (two times). Finally, the solid was dried in a vacuum oven at 40 °C for 24 h to give viscous PTFEA (*M*_n,SEC_ = 8800 g mol^−1^, *Đ* = 1.32). ^1^H NMR (400 MHz, CDCl_3_, ppm): 4.47 (2H, –OC*H*_2_CF_3_), 2.44 (H, –CH_2_–C*H*–), 1.58–2.04 (2H, –C*H*_2_–CH–)

#### 4.3.2. Post-polymerization reaction of PTFEA with amine

The typical procedure for the post-polymerization reaction of PTFEA with an amine is carefully described as follows: PTFEA (135 mg, 1 mmol, 1 equiv.), TA (69 mg, 1 mmol, 1 equiv.), DBU (456 mg, 3 mmol, 3 equiv.) and iso-propylamine (354 mg, 6 mmol, 6 equiv.) were added sequentially to a flask filled with 5 mL of acetonitrile (ACN). A portion of the mixture was withdrawn for the characterization to survey the reaction kinetics at different times, *e.g.*, ^1^H NMR and SEC. After the designated time, 10 mL dichloromethane (DCM) was added and the solution was extracted with 1 M HCl (aq.) 3 times. The organic phase was collected, dried with MgSO_4_ and then concentrated. The remaining mixture was poured into a large excess of *n*-hexane, decanted and dried in a vacuum oven at 40 °C for 24 h to give white solid poly(*N*-isopropylacrylamide) (PNIPAM). Other post-polymerization reactions with single amine were prepared in a similar procedure and the results are summarized in Table 1 and Fig. 2 (PNIPAM, *M*_n,SEC_ = 9400 g mol^−1^, *Đ* = 1.45). ^1^H NMR (400 MHz, CDCl_3_, ppm): 4.03 (H, –NH–C*H*–), 2.52 (H, –CH_2_–C*H*–), 2.08–1.59 (2H, –C*H*_2_–CH–), 1.33–1.16 (6H, –CH–(C*H*_3_)_2_).

#### 4.3.3. Post-polymerization modification of PTFEA with different amines

The typical process for the post-polymerization reaction of PTFEA with iso-propylamine and 1-cyclohexen-1-yl-ethylamine is carefully described herein: PTFEA (270 mg, 2 mmol, 1 equiv.), TA (138 mg, 2 mmol, 1 equiv.), DBU (912 mg, 6 mmol, 3 equiv.) and iso-propylamine (708 mg, 12 mmol, 6 equiv.) were added to a flask filled with 10 mL of ACN. After the designated time, the unreacted amine was expelled under reduced pressure. Subsequently, 1-cyclohexen-1-ylethylamine (756 mg, 6 mmol, 6 equiv. to PTFEA) was added to the flask at room temperature for 24 h. The reaction mixture was treated similarly to the method described above to give random copolymer poly(*N*-[(1-cyclohexen-1-yl)ethyl]acrylamide)_*x*_-*co*-PNIPAMy, Fig. 6), where *x* and *y* are the molar fractions of the amines in the attained polymers.^1^H NMR (400 MHz, CDCl_3_, ppm): 5.45–5.30 (H, –C=C*H*–), 4.03 (H, –NH–C*H*–), 3.32 (2H, –NH–C*H*_2_–CH_2_–), 2.10 (3H, –CH_2_–C*H*–, –NH–CH_2_–C*H*_2_–), 1.94–1.27 (10H, –C*H*_2_–CH–, –C(C*H*_2_–C*H*_2_–C*H*_2_–C*H*_2_–CH–).

## Conflicts of interest

The authors declare no conflict of interest.

## Supplementary Material
